# Prenatal earthquake stress exposure in different gestational trimesters is associated with methylation changes in the glucocorticoid receptor gene (NR3C1) and long-term working memory in adulthood

**DOI:** 10.1038/s41398-022-01945-7

**Published:** 2022-04-29

**Authors:** Ran Wang, Jincheng Wang, Shuqi Xu, Lan Wang, Mei Song, Cuixia An, Xueyi Wang

**Affiliations:** 1 Department of Psychiatry, The First Hospital of Hebei Medical University, Shijiazhuang, Hebei province People’s Republic of China; 2 Mental Health Centre of Hebei Medical University, Shijiazhuang, Hebei province People’s Republic of China; 3 Hebei Key Laboratory of Brain Science and Psychiatric-Psychologic Disease, Shijiazhuang, Hebei province People’s Republic of China

**Keywords:** Predictive markers, Clinical genetics

## Abstract

Prenatal stress exposure is thought to affect the long-term development of the foetal brain via the HPA axis and to change health outcomes in adulthood, including working memory (WM). The potential mechanism is that there is a critical period of brain development of the foetus, which is a result of selective adaptation to the external environment. The human glucocorticoid gene (NR3C1) is associated with memory and cognition. This study investigates the association between earthquake stress during pregnancy and CpG methylation of the NR3C1 exon 1_F_ promoter and its influence on working memory in adulthood. DNA methylation analysis using bisulfite sequencing PCR was quantified in 176 subjects who were exposed or not exposed to intrauterine earthquake and were divided into three groups based on the pregnancy trimester. The Hopkins Verbal Learning Test-Revised (HVLT-R) and Brief Visuospatial Memory Test-Revised (BVMT-R) were used to assess working memory performance. The methylated NR3C1 exon 1_F_ promoter of the prenatal earthquake exposure (PEE) group was significantly higher than that of the control group (CN). Analysis of subgroups indicated that the subjects in the second trimester of PEE group showed significantly higher methylation than those in the third trimester. Significantly low BVMT-R scores were detected in those who experienced prenatal earthquake in the second trimester of PEE group. Methylated CpG site 1 may play a critical role in contributing to lower BVMT-R scores in the second trimester in the PEE group, and may offer a potential epigenetic mechanism that links prenatal stress and long-term effects on working memory.

## Introduction

Adverse early life stress, including prenatal stress, is thought to change the developing brain and lead to long-lasting effects on emotion and cognitive function [1]. Many studies have reported that low-level chronic stress and severe stress or trauma during the foetal period may increase the risk of cardiovascular diseases [2] and mental disorders, such as depression [3] and schizophrenia [4, 5] in adulthood. Little is known about the long-term effects of prenatal earthquake exposure on alterations in cognitive function; notably, in most previous studies, multiple stressors were investigated.

The foetal period is a critical stage for the development of the brain, which is vulnerable to environmental influences that can cause long-lasting or even permanent alterations in brain functions associated with cognition in adulthood [6–8]. Some research has indicated a relationship between prenatal stress and the development of cognitive function. Entringer et al. found that prenatal stress-related low birth weight or small body size is associated with lower language scores and spatial and attention ability in childhood [9]. Studies of rodents and nonhuman primates have also shown that prenatal stress influences the cognitive performance of offspring in adulthood [10, 11]. The mechanisms leading to these results are unclear. Most studies have focused on hippocampal-related impairment of memory because the hippocampus has been revealed to be an important target for early life events [12–15], and little research has discussed the potential relevance between prenatal stress and prefrontal-dependent working memory performance. As another important component of cognitive function, working memory is thought to relate to higher cognitive performance, such as language, comprehension and reasoning [16, 17]. Muhammad et al. found that maternal stress results in decreased dendritic spine densities and changed synaptic circuits in the prefrontal cortex, which may lead to impaired function of learning and memory in the offspring of rodents [18]. Several human studies have also shown an association between adverse prenatal event exposure and prefrontal cortex-dependent working memory performance [19–21]. Our team has been studying the association between early-life stress and psychosomatic diseases [22–24]. In the current study, we focused on the effect of prenatal earthquake exposure on long-term working memory.

Many animal and human studies have revealed that the HPA axis system of the foetus is highly sensitive to alterations in the maternal environment [25]. The potential mechanism linking prenatal stress exposure to memory and cognitive outcomes later in life is proposed to be correlated with epigenetically mediated changes in expression of HPA axis-related genes [26, 27]. As one of the critical genes in the functioning of the HPA axis system, NR3C1 is considered an environmentally responsive biosensor that can regulate HPA axis-related gene expression and hormone production under stress exposure [28]. Meaney et al. studied the effects of changes in the caregiving environment on the DNA methylation status of NR3C1 in mammalians, and found that they regulate the activity through the feedback mechanism of the HPA axis [29]. Studies in rodents also have been documented in association with early maternal separation stress. Kember et al. found that male offspring exposed to early maternal separation exhibited behavioural inhibition in maze exploration, mediated by DNA hyper-methylation of the gene encoding for glucocorticoid receptors [30]. Meakin et al. found that increased placental methylation of NR3C1 in people exposed to prenatal adverse stress is associated with impaired cognitive function in their childhood [31]. Many other studies have also reported that NR3C1 epigenetic changes, particularly promoter 1_F_ CpG methylation, are related to hypoactivities of the prefrontal cortex and result in decreased learning and working abilities in humans [32].

Few studies have focused on the association between NR3C1 promoter 1_F_ CpG methylation in those who have experienced prenatal earthquake stress and cognitive function in adulthood. Based on previous data, we hypothesized that methylated NR3C1 exon 1_F_ promoter CpG sites may be potential biomarkers for predicting long-term working memory in women exposed to prenatal earthquake stress. In our current study, we detected the methylation status of early life adverse stress-related NR3C1 promoter 1_F_ CpG sites and tried to correlate the methylation changes in these CpG sites in people who experienced the 7.8 magnitude Tangshan earthquake in utero with their working memory abilities 38 years later.

## Material and methods

### Subjects

A total of 947 prenatal Tangshan earthquake-exposed subjects were investigated among workers from the Kailuan Mining Group. A total of 501 healthy controls were recruited among workers from the Shijiazhuang Steel Mill Group; Shijiazhuang is 424 km from Tangshan, and the people of these regions share similar living environments and customs.

The inclusion criteria for the current study were people born and raised in Tangshan or Shijiazhuang, Hebei Province, and people born between July 29, 1976 and April 28, 1977. All subjects were interviewed by two senior psychiatrists based on the Structured Clinical Interview for DSM-IV (SCID) to exclude mental disorders. To minimize the influences of confounding factors on NR3C1 methylation, exclusion criteria included history of adverse birth conditions, and medication use history, current chronic diseases (epilepsy, hypertension, thyroid diseases, diabetes, infection) and exposure to other serious traumatic events in addition to earthquakes. It is well known that both smoking and alcohol affect DNA methylation, subjects with a history of smoking and alcohol were specifically excluded. A large number of smokers and drinkers were excluded may be associated with their heavy manual work. Finally, 100 subjects who experienced earthquake stress and 76 healthy controls were included in this study.

Informed consent was obtained from the Ethics Committee of the First Hospital of Hebei Medical University (No. 2014005), and written informed consent in accordance with the Declaration of Helsinki was obtained from all of the subjects before enrolment (The clinical trial registration number: ChiCTR-OOC-15006542).

### Questionnaire, and psychological evaluation and assessment

The related information of the mothers burdened with earthquake stress was collected via questionnaire. We obtained such information from the parents of subjects or others with knowledge of the event about subjects (such as grandparents, uncles, aunts of subjects). Traumatic events were assessed using the Childhood Trauma Questionnaire (CTQ) and the Life Events Scale (LES). We also used the Hamilton Anxiety/Depressive Scale (HAMA/HAMD) to evaluate the current symptoms of anxiety/depression. The Structured Clinical Interview for DSM-V was used as the diagnostic standard.

### Evaluation of working memory

The Hopkins Verbal Learning Test-Revised (HVLT-R) [33] was used to evaluate the learning ability, immediate recall, and retention of verbal information. The Brief Visuospatial Memory Test-Revised (BVMT-R) [34] was used to evaluate learning efficiency, immediate recall, and delayed recall in visuospatial memory. The same test was executed three times for each subject, and total scores were used in the current study.

### NR3C1 promoter DNA methylation status

**Fig. 1:**
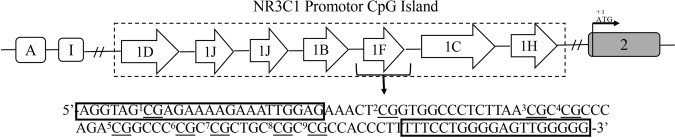
Analysed region of NR3C1 1_F_ promoter. The region from nucleotide −3184 to −3082 (numbers relative to the translation start site considered as +1) of the 5′- end of NR3C1 gene. Underlined: analysed CpG sites; square boxes: position of primers for amplification of bisulfite-treated DNA.

We collected blood samples in EDTA vacuum collection tubes (Inspeck, ST750EK, Sekisui, Osaka, Japan) at 8 a.m. in the morning. DNA was extracted from whole blood by a Gentra Puregene Blood Kit (Qiagen, Germantown, MD, USA). Genomic DNA samples (500 µg) were bisulfite-treated using the EZ DNA Methylation-Gold Kit (ZYMO, Irvine, CA, USA) following the manufacturer’s suggested protocol. We examined a portion of the exon 1 _F_ NR3C1 promoter, which was reported by Perroud et al., to show that adverse early life stress may permanently impact the HPA axis through epigenetic methylation of NR3C1 [35]. This portion contains nine CpGs that are located immediately downstream of those described as CpG39 by McGowan et al. (Fig. 1). The degree of each CpG site was analysed using BiQ Analyzer v2.0 software (http://biq-analyer.bioinf.mpi-inf.mpg.de). Primers for NR3C1 promoter PCR were designed using Primer 5.0 (forward: AGGTAGCGAGAAAAGAAATTGGAG; reverse: CCCCCAACTCCCCAAAAA). Primers were selected that covered a 102-bp region encompassing CpG sites 1–9, as reported by Perroud et al. [36]. The cycling conditions were 94 °C for 2 min followed by 100 cycles of 94 °C for 30 s and 65 °C to 55 °C Δ1 °C for 30 s with a final extension of 30 s at 72 °C. The PCR products for each sample were checked using a Prism 3730XL genetic analyser (Applied Biosystems, Foster, CA, USA).

### Statistical analyses

Two groups were compared using an unpaired t-test, and one-way ANOVA was performed to compare multiple groups. Fisher’s least significant difference test was used for post hoc analysis. The comparison of category data between groups was performed using a chi-square test. The Wilcoxon test was performed to compare nonnormally distributed data, and the Kruskal-Wallis test was used for comparisons among multiple groups. Pearson correlations were used to assess the association between the methylation status of the NR3C1 exon 1_F_ CpG promoter and the BVMT score at each trimester.

All methylation data were used without any transformation, as they were normally distributed according to the one-sample Kolmogorov-Smirnov test. To assess the association between maternal earthquake exposure and the methylation status of NR3C1, a fully adjusted regression model was used that included age, sex, and other potential covariates reported in previous studies, such as birth weight, childhood adverse event exposure, and current depressive/anxiety levels. To minimize the influences of confounding factors on cognition, a fully adjusted regression model also was used. Standardized effect size (Cohen’s effect size statistic) was used to calculate the difference in the means of the two groups. All of the data were analysed with SPSS 23.0 software, and the significance level was *p* < 0.05.

## Results

### Sample characteristics

This study included a total of 100 prenatal earthquake-exposed subjects and 76 no in uterus stress-exposed healthy controls. The subject characteristics are shown in Table 1. We compared the sociodemographic data and some relevant factors of NR3C1 methylation between the prenatal stress group and the healthy control group. Physical neglect based on the CTQ in the PEE group was significantly higher (*t* = 2.512, *P* = 0.011) than that in the CN group.

**Table 1:** Demographic characteristics.

	CN (*n* = 76)	PEE (*n* = 100)	*p* value
Age (years)	37.80 ± 0.97	38.14 ± 0.83	0.087
Gender			
Male	54	83	0.059
Female	22	17	
Birth weight (g)	3146.97 ± 405.84	3295.4 ± 891.40	0.179
Education (%)			
High school diploma or higher	64	74	0.103
Depressive/Anxiety symptom			
HAMD	1.79 ± 3.78	1.59 ± 2.88	0.143
HAMA	4.11 ± 5.12	2.23 ± 4.78	0.271
CTQ scores			
Emotional abuse	5.69 ± 1.17	5.48 ± 1.24	0.411
Emotional neglect	9.16 ± 3.90	9.63 ± 4.09	0.518
Sexual abuse	5.19 ± 0.63	5.10 ± 0.73	0.591
Physical abuse	5.31 ± 0.89	5.21 ± 0.70	0.093
Physical neglect	7.72 ± 2.19	8.38 ± 2.83	0.011^*^
Total CTQ	33.32 ± 6.42	33.84 ± 6.43	0.969
LES scores	43.43 ± 38.40	27.24 ± 28.33	0.051
Other trauma during pregnancy	None	None	—
Birth condition			
Birth asphyxia	None	None	—
Respiratory distress	None	None	—
Forceps delivery	None	None	—
Current chronic diseases	None	None	—
Psychiatry disease/symptom	None	None	—
Smoking	None	None	—
Alcohol	None	None	—
Medication use	None	None	—

*CN* Control Group, *PEE* Prenatal Earthquake Exposure Group, *HAMA* Hamilton Anxiety Scale, *HAMD* Hamilton Depression Scale, *CTQ* Childhood Trauma Questionnaire, *LES* Life Event Scale. Data are presented as mean ± SD; *p < 0.05.

### Effect of prenatal earthquake exposure on NR3C1 methylation

**Fig. 2:**
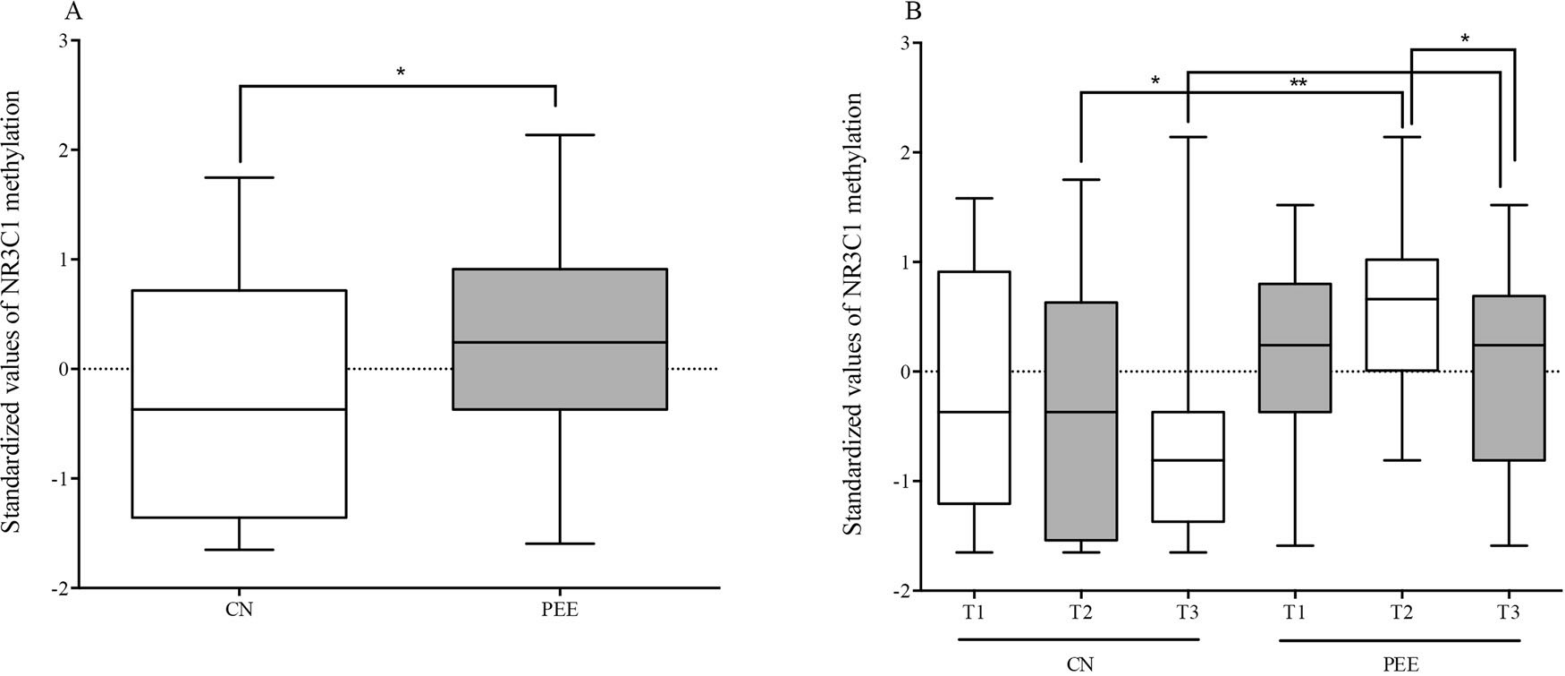
Box-plot of standardized methylated status in prenatal earthquake exposure and control groups. **A** A significant change in the methylated NR3C1 1 _F_ promoter was shown between the two groups. **B** The significantly different were detected across subgroups according to different trimesters of gestation when earthquake occurred. *CN* control group, *PEE* prenatal earthquake exposure group, *T1* first trimester, *T2* second trimester, *T3* third trimester. ^*^p < 0.05, ^**^p < 0.01.

There was a significantly higher standardized value of NR3C1 methylation in subjects who experienced prenatal earthquake exposure (*t* = 3.051; *P* = 0.000; [Cohen’s *d* = 0.57 SD units]) compared to the controls who did not experience intrauterine earthquake stress (Fig. 2B). After processing the effects of putative confounding factors (i.e., age, sex, birth weight, childhood stress exposure, current chronic stress, and depressive/anxiety levels), prenatal earthquake exposure was remarkedly associated with a higher NR3C11_F_ CpG methylation status (Supplementary Table S1). To further evaluate the effect of prenatal earthquake exposure on methylation status in adulthood, PEE subjects were divided into three subgroups, including the first (T1), second (T2), and third trimester (T3) groups, according to their gestational age when the earthquake occurred. After comparing each trimester, the result of a one-way ANOVA and *post hoc* analysis showed that the status of NR3C1 methylation in the second trimester (*t* = 3.315, *P* = 0.002) or third trimester (*t* = 2.124, *P* = 0.025) subgroups of the PEE group was significantly higher than that of the second trimester or third-trimester subgroups of the CN group, respectively. Further analysis showed a significant increase in NR3C1 methylation status in the PEE group during the second trimester (*t* = 2.145, *P* = 0.044) compared with the third trimester (Fig. 2B).

**Fig. 3:**
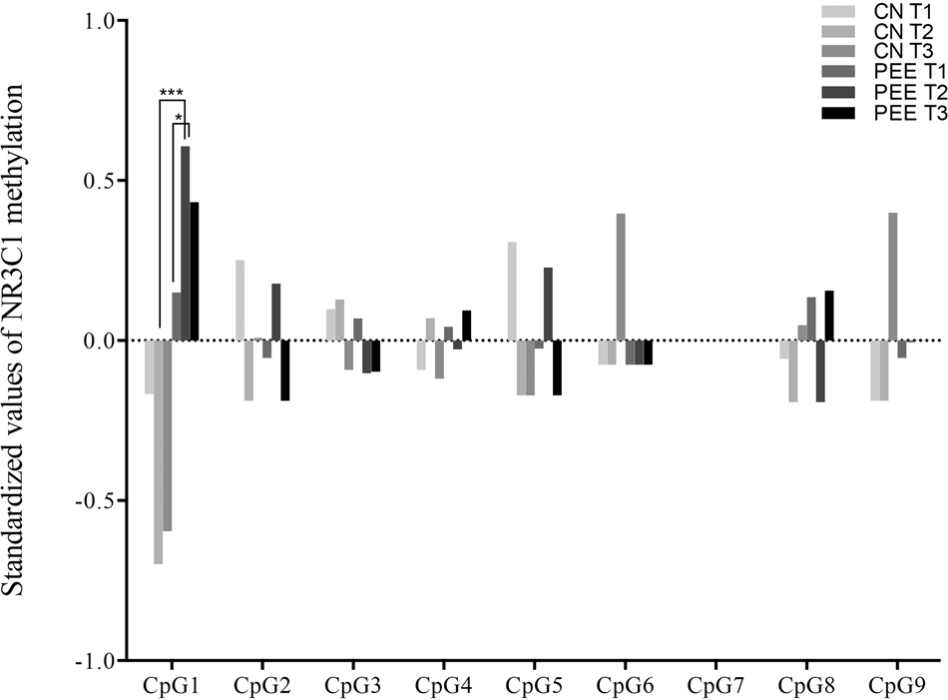
Standardized values of NR3C1 1_F_ promoter methylation in nine CpG sites of different subgroups. Only CpG1 site showed significantly change of methylation among the subgroup. The second trimester had a markedly higher methylation than the first trimester in PEE group. A significant higher methylated status also was detected in the second trimester of PEE group compared with the same subgroups of CN group. CN control group, PEE prenatal earthquake exposure group, *T1* first trimester, *T2* second trimester, *T3* third trimester. ^*^p < 0.05, ^***^p < 0.001.

After analysing each CpG site, we found that only the total methylated rate of whole subjects in the CpG1 site was more than 80%, and other sites with low methylation (less than 5%) or even no methylation were detected (Supplementary Fig. S1). We also assessed the different methylation rates at the CpG1 site in each subgroup. There was a markedly higher methylation status in the second trimester subgroup of the PEE group (*t* = 6.041, *P* = 0.000) than in the same subgroup of the CN group. Further analysis among the subgroups revealed that only the second trimester (*t* = 2.291, *P* = 0.035) had a significantly higher CpG 1 methylation status than the first trimester (Fig. 3).

### Comparison of working memory in the PEE and CN groups

**Fig. 4:**
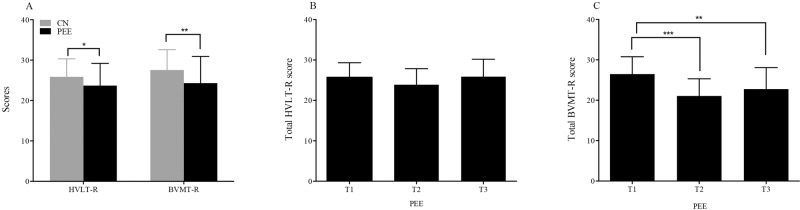
Box of total scores of HVLT-R and BVMT-R prenatal earthquake exposure and control groups. **A** The significant lower scores of both HVLT-R and BVMT-R were found between two groups. **B** No significant difference was found that compared the total BVMT-R scores among the subgroups of PEE group. **C** The second trimester of PEE group was showed a remarkedly lower compared to the first trimester or third trimester. *PEE* prenatal earthquake exposure group, *T1* first trimester, *T2* second trimester, *T3* third trimester, HVLT-R Hopkins Verbal Learning Test-Revised, BVMT-R Brief Visuospatial Memory Test-Revised. ^*^p < 0.05, ^*^^*^p < 0.01, ^*^^**^p < 0.001.

To assess visuospatial memory and verbal learning abilities, the BVMT-R and HVLT-R were performed on subjects in the PEE and CN groups. Test results were analysed and are shown in Fig. 4A. The total HVLT-R and BVMT-R scores in the PEE subjects (HVLT-R; *Z* = −5.477, *P* = 0.019, [Cohen’s *d* = 0.10 SD units]; BVLT-R; *Z* = −8.123, *P* = 0.004, [Cohen’s *d* = 0.47 SD units]) were significantly lower than those in the CN subjects (Supplementary Table S2). To further analyse the effect of prenatal earthquake stress on working memory in adulthood, we also compared the scores from HVLT-R and BVMT-R in the three subgroups. The BVMT-R scores differed markedly across the three subgroups, whereas the HVLT-R scores did not show significant changes (Fig. 4B & Supplementary Table S3). The BVMT-R scores in the subjects in the first trimester subgroup were significantly higher than those in the subjects in the second (*Z* = 20.850, *P* = 0.000) or third trimester subgroups (*Z* = 8.997, *P* = 0.003) of the PEE group (Fig. 4C). Although the scores from the second trimester were lower than those from the third trimester, there was no significant difference between the two subgroups. After processing the effects of potential confounding factors, only prenatal earthquake exposure was remarkedly associated with the scores of BVMT-R (Supplementary Table S4).

### Correlation between methylated CpG 1 of the NR3C1 exon 1 _F_ promoter and the BVMT-R score in the PEE group

**Fig. 5:**
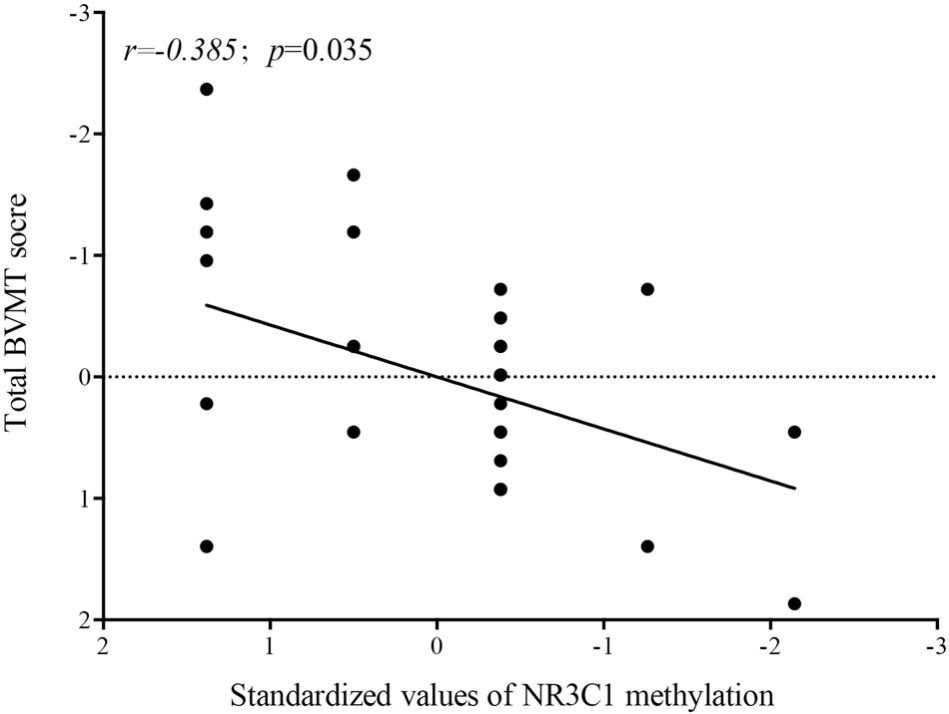
A Spearman correlation analysis for correlating methylated CpG 1 of NR3C1 exon 1 _F_ promoter and BVMT-R score in prenatal earthquake exposure group. A moderate negative correlation was found between methylated CpG1 site and BVMT-R scores only in the second trimester group. BVMT-R Brief Visuospatial Memory Test-Revised.

To estimate the influence of NR3C1 exon 1 _F_ CpG methylation on visuospatial memory in adults who were exposed to earthquake stress in utero, we performed a Pearson correlation analysis for the methylation status of CpG 1 and BVMT-R scores. The results showed that methylated CpG1 had a moderate negative correlation (*r* = −0.385, *P* = 0.035) with BVMT-R scores only in the second trimester group (Fig. 5).

## Discussion

Many studies have shown that the status of NR3C1 promoter methylation in the peripheral blood is associated with adverse early life events. Our previous studies also found that prenatal earthquake exposure was associated with health-related indicators (e.g., systolic blood pressure, BMI and low-density lipoprotein level) in adulthood [2]. However, few studies have reported the relationship between NR3C1 promoter methylation and cognitive function in adults who experienced earthquake stress in utero. Here, we report the results of the first study assessing the status of NR3C1 promoter methylation in subjects who experienced an earthquake intrauterine; we found significantly higher methylation levels in these subjects compared with subjects with no prenatal earthquake exposure. Further analysis of the results based on the gestational ages of the subjects at the time of the earthquake revealed an increased methylation status of subjects whose mothers were in the second or third trimester of pregnancy. These results did not change after controlling for several potential confounding factors. These findings suggest that a higher NR3C1 methylation status in adulthood is associated with earthquake stress exposure during the second or third trimester of pregnancy. Moreover, the increased methylation status is more obvious in the second trimester.

“Foetal programming” describes alterations in the development of the foetal brain due to environmental factors [37]. Cortisol, for instance, plays a crucial role in foetal development after exposure to maternal stress [38, 39]. Some studies found that the timing of foetal exposure to stress hormones and found that increased maternal cortisol at the second trimester was associated with decreased infant neuromuscular maturation, which may lead to preterm birth, low birth weight and an increased risk of schizophrenia [40, 41]. Based on our current results, we hypothesized that prenatal earthquake exposure in the second trimester of pregnancy compared with the third trimester may lead to alterations in GR expression via increased CpG methylation of the NR3C1 exon 1 _F_ promoter, which magnifies the negative effects of stress. In addition, it is noteworthy that in the comparison of sociodemographic data, we found that physical neglect score in PEE group was significantly higher than that in CN group. A large number of studies have also shown that early life events will change the HPA axis-related epigenetic markers (such as DNA methylation), which even might persist into adulthood and affect the vulnerability of psychopathology through effects on intermediate level of gene expression. Although in the current study, fully adjusting for physical neglect did not affect the level of NR3C1 methylation, the superposition of intrauterine stress and early caregiver behaviour on the changes of epigenetic markers, even HPA axis related function in adulthood cannot be completely ignored.

Gene epigenetic changes in DNA methylation status of the NR3C1 by maternal stress are thought to be primarily responsible for the increased sensitivity of the foetal HPA axis [29, 42]. Most human and animal studies have shown that HPA axis activity is associated with cognitive performance [43, 44]. DNA methylation of HPA axis-related genes may bridge HPA activity to cognitive function [45]. In current results, the BVMT-R scores of the subjects exposed to prenatal earthquake stress were significantly lower than those of the non-exposed subjects. After analysing the subgroups, the second-trimester subjects showed the worst performance in visuospatial memory compared with that the subjects of the other trimesters in the PEE group. Numerous studies have established that the foetal brain develops rapidly in the second trimester of gestation, at which point the placental CRH feedback circuit tends to mature. Intrauterine exposure may lead to a reduced neuronal content in the developing prefrontal cortex in this stage, which causes a long-term effect on PFC-dependent working memory in offspring [46]. BVMT-R displayed high sensitivity and specificity for visual learning and memory and delayed recall. A fMRI study documented that PFC activity was associated with performance on total BVMT-R scores and delayed recall, which highlights the importance of PFC mechanisms in working memory [47] and supports our current conclusions. The verbal learning and memory assessed by HVLT-R maybe more related to hippocampal-dependent function [48].

Keller et al. investigated the association of HPA axis activity, cortisol, clinical mood symptoms, and genetic variation with cognitive function and suggested that the NR3C1 genetic status was implicated in attention and working memory [49]. GR can affect learning and memory function by influencing neural structure integration, synaptic transmission efficiency and LTP formation, which is the basis of memory formation [50]. Huang et al. found that methylation alteration in key regions of NR3C1 may lead to offspring’s long-term cognitive impairment by early life-stress events [51]. However, few studies have investigated an association between the NR3C1 methylation status and long-term cognitive function in adulthood of the subjects who experienced the prenatal-earthquake stress. In our present study, Pearman correlations revealed that a lower BVMT-R score in adulthood was associated with a higher methylation status of NR3C1 exon 1 _F_ promoter CpG1 site in subjects exposed to prenatal earthquake stress in the second trimester. Our findings suggested that higher methylation in key regions of the NR3C1 promoter may bridge the intrauterine earthquake stress exposure and long-term effect on the ability of visuospatial memory in offspring. The methylation status of CpG1 would be a potential biomarker for predicting the long-term PFC-dependent working memory in people who experienced prenatal stress.

A limitation of this study is that we used another city as the control group; this city is in the same province as Tangshan, and the people of both regions share similar living environments and customs. Although we performed a fully adjusted regression to evaluate some reported potential confounding factors that may affect NR3C1 promoter methylation, some limitations could not be ruled out such as parenting and family condition. In order to further clarify the effect of NR3C1 methylation on earthquake-stress intrauterine and BVMT-score, we used the status of NR3C1 methylation as mediators and performed the mediation analysis, while insignificant mediation effect was found (data not show). This may be due to the insufficient sample size and/or numerous potential factors mediating prenatal stress and BVMT-R score in adulthood. Thereby, we should focus on the research of potential factors, especially the influence of multi-gene interaction on early-life stress and cognitive function in the future. In spite of this, from the current results we also consider it can be used as the first step of this hypothesis. Several studies have reported the sex-specific effects on stress-related cognitive function. We could not examine the potential sex difference because the occupational characteristics of the subjects in this study caused a great difference between the sexes. More detailed information and larger sample sizes should be collected in further work to improve the current results. Another limitation is lack of measures of HPA axis-related hormones. This limitation is due to the lack of sufficient research conditions during the experiment, which make it impossible for us to improve and explain the current hypothesis. In future research, we plan to carry out follow-up study and HPA axis measurement to supplement the inadequacy of the present study.

## Supplementary information

Supplementary Figure S1 Legend

Supplementary Figure S1

Supplementary Table S1

Supplementary Table S2

Supplementary Table S3

Supplementary Table S4
