# Supplementary material for: Prenatal earthquake stress exposure in different gestational trimesters is associated with methylation changes in the glucocorticoid receptor gene (NR3C1) and long-term working memory in adulthood
Source: Transl Psychiatry. 2022 Apr 29;12:176. doi: 10.1038/s41398-022-01945-7 (PMC9054818; doi:10.1038/s41398-022-01945-7)
Supplement: Supplementary file 1 — Supplementary Figure S1 Legend [file 41398_2022_1945_MOESM1_ESM.docx]

**Supplementary Fig.S1 Total methylated rate of whole sample in nine CpG sites.** Only the total methylated rate of whole subjects in the CpG1 site was more than 80%, and other sites with low methylation (less than 5%) or even no methylation were detected.
