# Supplementary figures and images for: Prenatal earthquake stress exposure in different gestational trimesters is associated with methylation changes in the glucocorticoid receptor gene (NR3C1) and long-term working memory in adulthood
Source: Transl Psychiatry. 2022 Apr 29;12:176. doi: 10.1038/s41398-022-01945-7 (PMC9054818; doi:10.1038/s41398-022-01945-7)

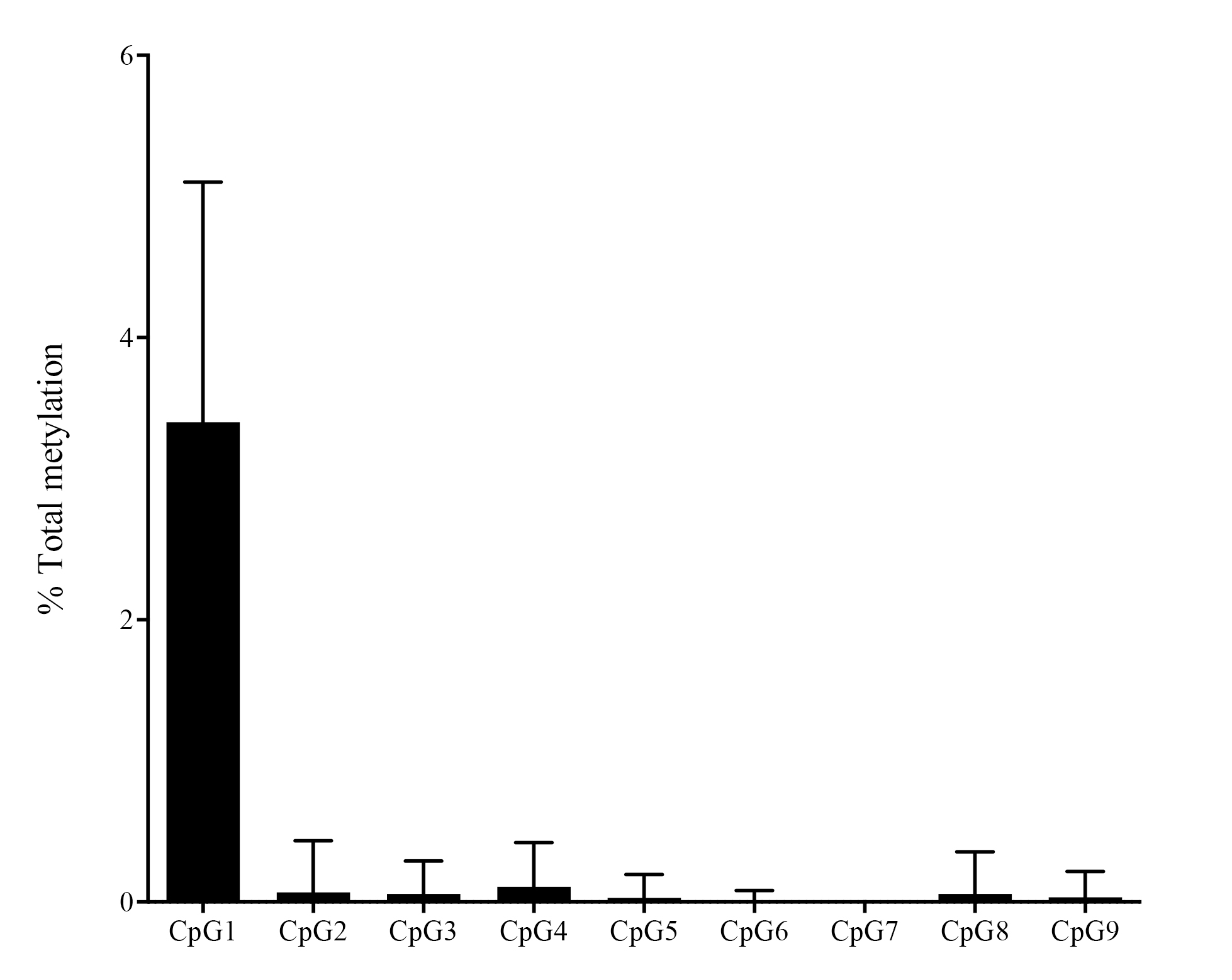

Supplement: Supplementary file 2 — Supplementary Figure S1 [file 41398_2022_1945_MOESM2_ESM.jpg]
