# Supplementary material for: Prenatal earthquake stress exposure in different gestational trimesters is associated with methylation changes in the glucocorticoid receptor gene (NR3C1) and long-term working memory in adulthood
Source: Transl Psychiatry. 2022 Apr 29;12:176. doi: 10.1038/s41398-022-01945-7 (PMC9054818; doi:10.1038/s41398-022-01945-7)
Supplement: Supplementary file 3 — Supplementary Table S1 [file 41398_2022_1945_MOESM3_ESM.docx]

Supplementary Table S1. Fully adjusted regression of putative confounding factors for NR3C1 methylation

|  | Unstandardized β(95% CI) | | |
| --- | --- | --- | --- |
|  | Whole | Male | Female |
| Age | -0.185(-0.927~0.557) | -0.202(-1.005~0.600) | 0.094(-2.613~2.8010） |
| Gender  Birth weight | -0.312(-1.170~0.547)  0.319(-0.274~0.912) | -  0.278(-0.377~0.933) | -  0.523(-0.882~1.928) |
| Prenatal earthquake exposure | -1.178(-1.183~-0.472)** | -0.996(-1.784~ -0.207)* | -2.295(-4.479~ -0.111)* |
| CTQEA | 0.635(-0.164~1.433) | 0.408(-0.449~1.264) | 3.927(-0.559~8.412) |
| CTQEN | -0.609(-1.517~0.298) | -0.861(-1.876~ 0.155) | -0.099(-3.046~2.849) |
| CTQSA | -0.167(-1.241~0.90) | -0.272(-1.406~0.863) | 2.146(-4.070~8.363) |
| CTQPA | -0.873(-1.967~0.220) | -0.734(-1.922~0.454) | -0.786(-4.830~3.257) |
| CTQPN | -0.271(-1.088~0.546) | -0.047(-0.940~0.846) | -1.049(-3.706~1.608) |
| CTQT | 0.819(-0.238~1.877) | 0.945(-0.210~2.099) | -0.361(-3.643~2.921) |
| LES | -0.115(-0.810~0.581) | 0.060(-0.721~0.842) | -1.928(-4.850~0.994) |
| HAMA | 0.140(-0.807~1.086) | 0.669(-0.467~1.806) | -2.914(-6.481~0.653) |
| HAMD  Education | 1.027(-0.493~2.547)  0.420(-0.201~1.017) | 0.612(-1.421~2.644)  0.302(-0.395~1.075) | 4.499(-0.236~9.234)  0.154(-0.989~0.646) |

HAMA, Hamilton Anxiety Scale; HAMD, Hamilton Depression Scale; LES, Life Event Scale. *p < 0.05; **p < 0.01.
