# Supplementary material for: Prenatal earthquake stress exposure in different gestational trimesters is associated with methylation changes in the glucocorticoid receptor gene (NR3C1) and long-term working memory in adulthood
Source: Transl Psychiatry. 2022 Apr 29;12:176. doi: 10.1038/s41398-022-01945-7 (PMC9054818; doi:10.1038/s41398-022-01945-7)
Supplement: Supplementary file 4 — Supplementary Table S2 [file 41398_2022_1945_MOESM4_ESM.docx]

Supplementary Table S2

| Scores | Control | Prenatal earthquake stress | *Z* | *P* |
| --- | --- | --- | --- | --- |
| HVLT-R | 25.85±4.47 | 23.71±5.48 | 5.477 | 0.019* |
| BVMT-R | 27.11±5.12 | 24.32±36.59 | 8.123 | 0.004** |
