# Supplementary material for: Prenatal earthquake stress exposure in different gestational trimesters is associated with methylation changes in the glucocorticoid receptor gene (NR3C1) and long-term working memory in adulthood
Source: Transl Psychiatry. 2022 Apr 29;12:176. doi: 10.1038/s41398-022-01945-7 (PMC9054818; doi:10.1038/s41398-022-01945-7)
Supplement: Supplementary file 5 — Supplementary Table S3 [file 41398_2022_1945_MOESM5_ESM.docx]

Supplementary Table S3

| Scores | First Trimester | Second trimester | Third trimester | *H* | *P* | *Z^a^* | *P^a^* | *Z^b^* | *P^b^* | *Z^c^* | *P^c^* |
| --- | --- | --- | --- | --- | --- | --- | --- | --- | --- | --- | --- |
| HVLT-R | 25.68±3.64 | 23.90±3.69 | 25.86±4.31 | 5.626 | 0.06 | - | - | - | - | - | - |
| BVMT-R | 26.49±4.28 | 21.07±4.25 | 22.76±5.32 | 22.158 | <0.000 | 20.850 | <0.000 | 8.997 | 0.003** | 1.651 | 0.199 |
