# Supplementary material for: Prenatal earthquake stress exposure in different gestational trimesters is associated with methylation changes in the glucocorticoid receptor gene (NR3C1) and long-term working memory in adulthood
Source: Transl Psychiatry. 2022 Apr 29;12:176. doi: 10.1038/s41398-022-01945-7 (PMC9054818; doi:10.1038/s41398-022-01945-7)
Supplement: Supplementary file 6 — Supplementary Table S4 [file 41398_2022_1945_MOESM6_ESM.docx]

Supplementary Table S4. Fully adjusted regression of putative confounding factors

for BVMT-R and HVLT-R

|  | Unstandardized β(95% CI) | |
| --- | --- | --- |
|  | BVMT-R | HVLT-R |
| Age | 0.192(0.569~2.582) | -0.439(0.309~1.346) |
| Gender  Birth weight | -0.772(0.152~1.405)  -0.548(0.300~1.113) | -0.008(0.343~2.870)  -0.350(0.376~1.321) |
| Prenatal earthquake exposure | -1.208(0.003~0.299)^**^ | 0.361(0.664~2.831) |
| CTQEA | 0.559(0.745~4.104) | -0.254(0.356~1.264) |
| CTQEN | -0.760(0.182~1.204) | -0.820(0.179~ 1.082) |
| CTQSA | -0.315(0.244~2.186) | -0.809(0.154~1.287) |
| CTQPA | -1.118(0.103~1.040) | -0.002(0.353~2.843) |
| CTQPN | -0.985(0.161~1.865) | -0.613(0.242~1.213) |
| CTQT | 0.343(0.482~4.115)) | 0.927(0.898~7.117) |
| LES | 0.170(0.583~2.411) | 0.259(0.653~2.572) |
| HAMA | 0.015(0.370~2.790) | -0.163(0.327~2.202) |
| HAMD  Education | 0.258(0.277~6.045)  0.170(0.482~2.916) | -0.026(0.223~4.263)  -0.027(0.409~2.318) |

HAMA, Hamilton Anxiety Scale; HAMD, Hamilton Depression Scale; LES, Life Event Scale.

**p < 0.01.
